# Friedreich’s Disease

**Published:** 1906-07

**Authors:** Wesley E. Taylor


### FRIEDREICH'S DISEASE,

BY WESLEY E. TAYLOR, M.D.

This disease was first described by Duchenne, of Boulogne, and later by Friedreich in 1861, though it was not accepted as a separate and distinct pathological entity till some time in the 80's.

The disease exists probably from the birth of the patient, but manifests itself first, as a rule, at the time of puberty. Heredity plays the principal etiological role, and a nervous tare can always be found in the ancestors. Alcohol and syphilis, as well as trauma and the infectious fevers, are very questionable factors indeed. At the time of puberty the first symptoms begin to be observed as a weakness in the lower limbs, associated with incoordination and a certain oscillatory unsteadiness in the bodily movements. This ataxia increases and gradually extends upward, involving the musculature of the trunk, upper limbs, speech organs, eyes and head in turn. Nearly all the symptoms of this disease are due to this choreiform instability of the musculature. Among the less constant symptoms are a greater or lesser degree of kyphoscoliosis and a peculiar deformity of the foot—a "foot drop," which, while not constant, is very characteristic. The trouble with the gait [illegible]mbles very closely the unsteadiness of the drunkard in its oscillations. When sitting the body sways and the head rocks backward and forward in a most uncertain and unrythmical manner. In attempting to grasp an object, the motion made is comparable to that of a bird of prey. There is an oscillating and unsteady swaying motion as the hand approaches the object—then a momentary pause, as if balancing, followed by a swoop down upon the object, and with fairly certain aim, too, when it is grasped without much difficulty.

The development of the disease is not rapid, as the lower limbs may be affected several years before it extends to the arms and head. The muscular strength suffers but little, especially in the earlier stages, though the muscular contractility is slower than normal. Facial grimaces are common, as well as fibrillary twitchings of the tongue, and it is the ataxia of the tongue which produces the hesitating ("scanning") speech, though mastication and deglutition remain normal. This choreiform instability of most of the muscles is admirably well observed in the unsteadiness of the eyes, where, when the patient fixes even in front, an irregular nystagmus can be seen. The tendinous reflexes disappear early in the course of the disease, and there is usually a hypotonus of the muscles. Sensory changes are rarely observed, though some vague pains and formications, as well as occasional vertigo and headaches, are not unusual symptoms. The sexual powers are delayed, but when they do appear seem to be normal. The intelligence is unaffected. The clinical picture is usually complete in six or eight years, and at twenty-five or thirty there is complete bodily impotence, and the patient is confined to bed unable to help himeslf. The disease does not seem to be fatal, because the patient succumbs as a rule to some intercurrent disease. A cure has never been observed. Pathologically, the affection is one of the spinal cord—a developmental imperfection affecting the posterior columns—usually irregular, though symmetrical. Morphologically, it is a sclerosis of the neurogliar tissue. Some think that this sclerosis has its origin in a circulatory abnormality, though there is considerable diversity of opinion.

*Diagnosis.*—Freidreich's ataxia can be differentiated from *hereditary tabes* by the Argyle-Robertson pupil, the pains, the sensory and visceral troubles. From *multiple sclerosis* by the increased reflexes, the contracture, the intentional tremor, though ataxia, speech troubles and nystagmus are common to both.

From *hereditary cerebral ataxia* by the optic atrophy, the Argyle-Robertson pupil and the diminished visual field.

From *interstital hypertrophic neuritis* by the muscular atrophy, sensory troubles, hypertrophy of nerve trunks, Argyle-Robertson pupil and Romberg's symptom. *Cerebellar tumors* must also be remembered and excluded.

My case is that of a little waif of nine years, whose ancestry and previous history is unknown. She has absence of reflexes, though by having her pull her hands we can get a slight knee jerk. The speech difficulties are characteristic, though not at all times of the same intensity. The awkwardness is variable, but the ataxia typical. She has occasional pains in arms and limbs, and sometimes dizziness. Kyphoscoliosis is quite noticeable. There are no sensory disturbances, mental deficiency or imperfections of development.

